# Inflammation and Gli2 Suppress Gastrin Gene Expression in a Murine Model of Antral Hyperplasia

**DOI:** 10.1371/journal.pone.0048039

**Published:** 2012-10-24

**Authors:** Milena Saqui-Salces, Evelyn Covés-Datson, Natalia A. Veniaminova, Meghna Waghray, Li-Jyun Syu, Andrzej A. Dlugosz, Juanita L. Merchant

**Affiliations:** 1 Department of Internal Medicine, University of Michigan, Ann Arbor, Michigan, United States of America; 2 Department of Dermatology, University of Michigan, Ann Arbor, Michigan, United States of America; 3 Cell and Developmental Biology, University of Michigan, Ann Arbor, Michigan, United States of America; 4 Molecular and Integrative Physiology, University of Michigan, Ann Arbor, Michigan, United States of America; Indiana University School of Medicine, United States of America

## Abstract

Chronic inflammation in the stomach can lead to gastric cancer. We previously reported that gastrin-deficient (*Gast^−/−^*) mice develop bacterial overgrowth, inflammatory infiltrate, increased Il-1β expression, antral hyperplasia and eventually antral tumors. Since Hedgehog (Hh) signaling is active in gastric cancers but its role in precursor lesions is poorly understood, we examined the role of inflammation and Hh signaling in antral hyperplasia. *LacZ* reporter mice for *Sonic hedgehog* (*Shh*), *Gli1*, and *Gli2* expression bred onto the *Gast^−/−^* background revealed reduced *Shh* and *Gli1* expression in the antra compared to wild type controls (WT). *Gli2* expression in the *Gast^−/−^* corpus was unchanged. However in the hyperplastic *Gast^−/−^* antra, *Gli2* expression increased in both the mesenchyme and epithelium, whereas expression in WT mice remained exclusively mesenchymal. These observations suggested that *Gli2* is differentially regulated in the hyperplastic *Gast^−/−^* antrum versus the corpus and by a Shh ligand-independent mechanism. Moreover, the proinflammatory cytokines Il-1β and Il-11, which promote gastric epithelial proliferation, were increased in the *Gast^−/−^* stomach along with Infγ. To test if inflammation could account for elevated epithelial *Gli2* expression in the *Gast^−/−^* antra, the human gastric cell line AGS was treated with IL-1β and was found to increase *GLI2* but decrease *GLI1* levels. IL-1β also repressed human *GAST* gene expression. Indeed, GLI2 but not GLI1 or GLI3 expression repressed gastrin luciferase reporter activity by ∼50 percent. Moreover, chromatin immunoprecipitation of GLI2 in AGS cells confirmed that GLI2 directly binds to the *GAST* promoter. Using a mouse model of constitutively active epithelial GLI2 expression, we found that activated GLI2 repressed *Gast* expression but induced *Il-1β* gene expression and proliferation in the gastric antrum, along with a reduction of the number of G-cells. In summary, epithelial Gli2 expression was sufficient to stimulate *Il-1β* expression, repress *Gast* gene expression and increase proliferation, leading to antral hyperplasia.

## Introduction

The two histologically and physiologically distinct compartments of the mouse glandular gastric epithelium are: the proximal corpus/fundus (oxyntic) mucosa characterized by the presence of acid-producing parietal cells, and the distal endocrine mucosa (antrum) composed of enteroendocrine cells (G cells) that secrete the hormone gastrin (Gast) [1]. Gast stimulates the parietal cells in the corpus to secrete acid. In addition, the hormone is considered to be a growth factor for the gastrointestinal tract [2], [3], and on that basis has been implicated in gastrointestinal cancers [4], [5].

In the normal gastric corpus, Hedgehog (Hh) ligands such as Sonic hedgehog (Shh) are produced, but then decrease with chronic inflammation, loss of acid secretion (hypochlorhydria), which leads to gastric metaplasia, a precursor lesion for gastric cancer [6], [7], [8]. Nevertheless, Hh signaling remains active in gastric cancers [9], suggesting differences in the regulation of the Hh pathway in normal stomach compared to gastric carcinogenesis. We and others have analyzed the role of Hh signaling in the gastric corpus [6], but information on Hh signaling in the gastric antrum and its participation in antral tumor formation is scarce. In addition, Shh, the major Hh ligand expressed in the corpus, subsequently diminishes in the distal stomach (antrum) despite persistent expression of Hh gene targets, e.g., Gli1 and Gli2 [10], [11], [12], suggesting differential Hh signaling pathways operating in these two regions of the stomach.

Gastric cancer is among the more prevalent cancers worldwide, with a survival rate of 27% [13]. Interestingly, a shift in the most frequent site of gastric cancer from the distal stomach (antrum) to the more proximal corpus and cardia has been observed over the past 10 years, possibly reflecting differences in cancer etiology and risk factors for these two regions of the stomach [14]. Mouse models of gastric tumorigenesis frequently exhibit changes in the gastric corpus/fundus with little or no changes in the antrum. However to accurately compare the etiologic differences in cancer development between these two anatomic sites, further dissection of the mechanisms leading to hyperplasia and eventually tumorigenesis in the antrum is needed. Currently, different genetic models of antral cancer have been described and include loss of trefoil factor 1 (TFF1) [15], aberrant activation of the gp130 cytokine receptor [16] and loss of the hormone gastrin (*Gast^−/−^)* [17], yet none have examined a specific role for Hh signaling.

In a previous study, we reported that increased expression of Il-1β, and the Tgfβ- family members activin A (AcA) and follistatin (Fst) precede gastric transformation in the *Gast^−/−^* mice [18]. Tumors in this model occur when mice are older than 9 months and their development has been associated with bacterial overgrowth [19] and inflammation [20], [21]. By the time antral tumors are detected, mice may have also developed corpus atrophy due to hypochlorhydria [18], [20]. Therefore to better define the changes that are associated with the initiation of antral tumors, we analyzed *Gast^−/−^* mice between 9 and 13 months of age, which showed only antral hyperplasia without obvious histological changes in the corpus, for Hh signaling. We report here that *Gli2* was induced in early antral lesions in a Shh-independent manner. Moreover, proinflammatory cytokines increased along with proliferative indicators while *Gast* gene expression decreased.

**Figure 1:**
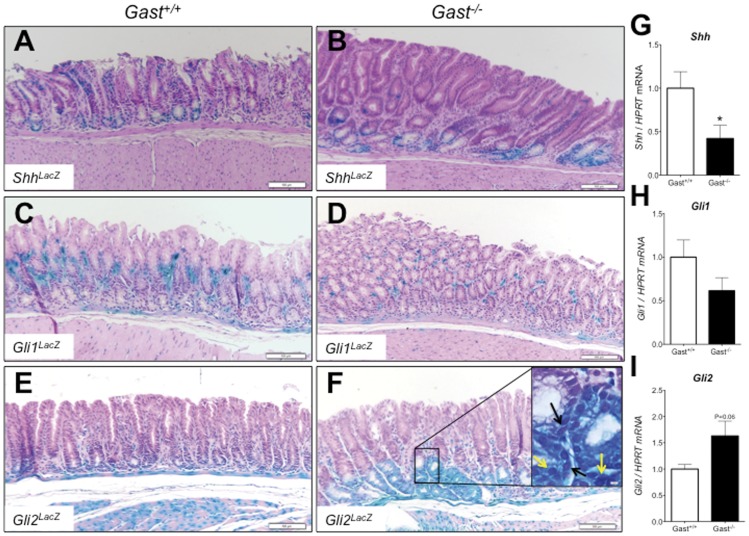
Epithelial expression of Gli2 in the *Gast^−/−^* antrum. The antral expression of Hedgehog pathway molecules was determined in 9–13 month-old littermate controls (*Gast^+/+^*) (panels **A**, **C** and **E**) and *Gast^−/−^* (panels **B**, **D** and **F**) mice by X-gal staining of LacZ reporter mice for Sonic hedgehog (*Shh*) (**A** and **B**), *Gli1* (**C** and **D**) and *Gli2* (**E** and **F**). A high power field of Gli2-LacZ staining is shown in F, where nuclear (yellow arrows) and perinuclear (black arrows) staining was observed along with cytoplasmic reporter accumulation. Whole stomachs from *Gast^+/+^* and *Gast^−/−^* were analyzed for gene expression of *Shh* (**G**), *Gli1* (**H**), and *Gli2* (**I**). Bars in panels **A** to **F** are 100 µm. Data presented as mean±SEM. N = 8 per group. *P≤0.05.

## Methods

### Ethics Statement

All animal procedures were approved by the University of Michigan Animal Care and Use Committee (DHHS Animal Welfare Assurance A3114-01).

### Animals

Gastrin deficient (*Gast^−/−^*) mice [17], [18] were bred to mice carrying the bacterial β-galactosidase (*lacZ*) gene that was either inserted, together with an IRES element into the 3′ untranslated region of the *Shh* gene (*Shh^lacZ^*) [22], or disrupted one *Gli1* (*Gli1^lacZ^*) or *Gli2* (*Gli2^lacZ^*) [23] allele. Animals were conventionally housed in microisolator cages in nonbarrier mouse rooms.

**Figure 2:**
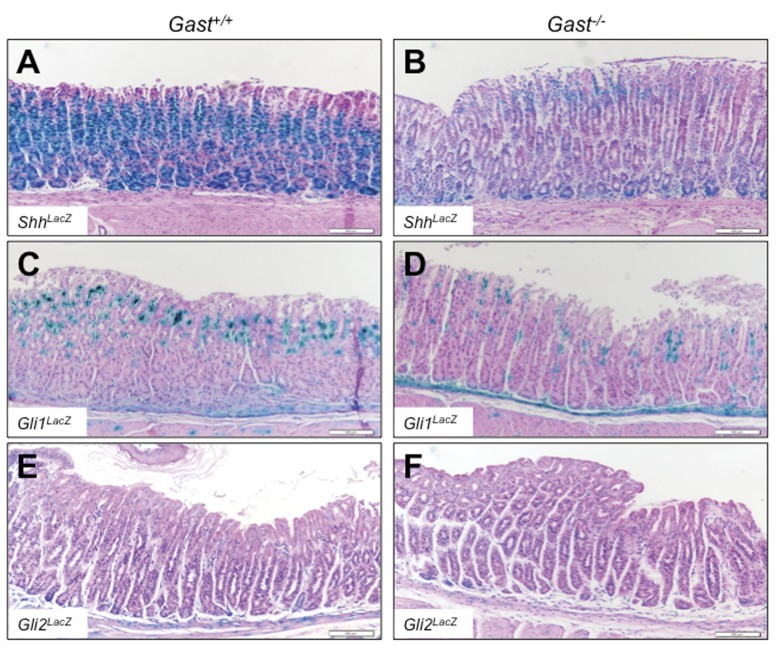
Gli2 expression is not increased in the *Gast^−/−^* corpus. Representative X-gal staining of corpi of *Gast^+/+^* and *Gast^−/−^* mice harboring the *LacZ* reporter for Sonic hedgehog (*Shh*) (**A** and **B**), *Gli1* (**C** and **D**) and *Gli2* (**E** and **F**). Bars are 100 µm.

### Inducible GLI2 Transgene Expressing Mice

To activate GLI2 expression *in vivo*, we generated a doxycycline-inducible mouse model carrying a MYC-tagged, activated form of GLI2, designated GLI2?N. Generation of *Shh-Cre;R26-LSL-rtTA;tetOGLI2ΔN* triple allele transgenic mice has been previously described [24], [25]. This model utilizes mice carrying 3 alleles: (a) a tissue-specific Cre driver (*Shh-Cre*); (b) the Cre-inducible *R26-LSL-rtTA* strain [25]; and (c) a *tetO-GLI2*Δ*N*. Mice were bred according to standard protocols to generate triple-transgenic mice. To induce transgene expression, mice were fed chow containing 1 g doxycycline/kg chow (Bio-Serv, Frenchtown, NJ) and 200 µg/ml doxycycline (Sigma-Aldrich, St. Louis, MO) in their drinking water with 5% sucrose.

### X-gal Staining

β-galactosidase activity (LacZ) was detected in whole stomach X-gal staining as previously described [6]. Briefly, stomachs were fixed in 4% buffered paraformaldehyde for 1 h at 4°C, washed in phosphate-buffered saline (PBS) with 0.01% sodium deoxycholate and 0.02% NP-40), and then stained overnight at 4°C with 1 mg/ml of the X-gal substrate in fresh 5 mM potassium ferrocyanide and 5 mM potassium ferricyanide. After staining, samples were washed and post-fixed in 4% buffered formaldehyde and processed for paraffin embedding.

**Figure 3:**
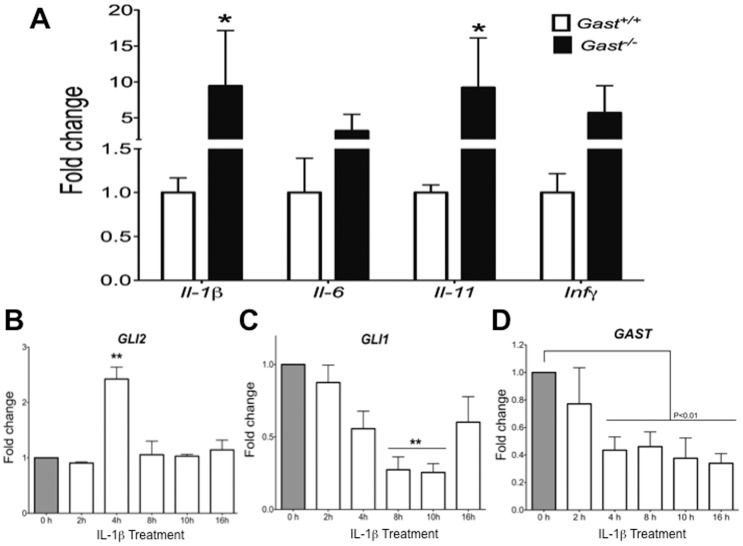
IL-1β induced *Gli2*, but not Hh signaling in gastric epithelial cells. **A**) Gene expression of inflammatory cytokines *IL-1β*, *IL-6*, *IL-11* and *INFγ* were determined in the stomachs of *Gast^+/+^* and *Gast^−/−^* mice by qRT-PCR. The gastric cell line AGS was treated with 0.1 ng/ml of IL-1β for different time points and their gene expression of *GLI2* (**B**), *GLI1* (**C**) and *GAST* (**D**) were measured. Data presented as mean ± SEM. N = 8 per group in panel **A**. **B**, **C** and **D**: N = 3 independent experiments. *P≤0.05, **P≤0.01.

### Immunohistochemistry and Immunofluorescence

Stomachs were fixed in 4% buffered formaldehyde and paraffin-embedded. Longitudinal sections (5 µm) were deparaffinized and antigen retrieval was performed using by boiling the slides in 10 mM sodium citrate buffer, pH 6 for 40 min. Rabbit anti-gastrin (Dako, Carpinteria, CA), rabbit anti-Ki-67 (Thermo Scientific, Fremont, CA), rabbit anti-MYC (Cell Signaling, Danvers, MA). Donkey antibodies conjugated to Alexa-488 or Alexa-594 (Jackson ImmunoResearch Laboratories, West Grove, PA) were the secondary antibodies used to detect the primary antibody by immunofluorescence. Nuclei were counterstained with 4,6-diamidino-2-phenylindole dihydrochloride (DAPI).

### Cell Culture

The gastric cancer cell lines AGS and NCI-N87 were purchased from the American Type Culture Collection (ATCC, Manassas, VA) then grown to 80% confluence in RPMI-1640 media supplemented with 10% FBS and 1% antibiotics. Following serum starvation for 24 h, cells were treated with IL-1β (0.1 ng/ml, R&D Systems, Minneapolis, MN) in serum-free culture medium.

**Figure 4:**
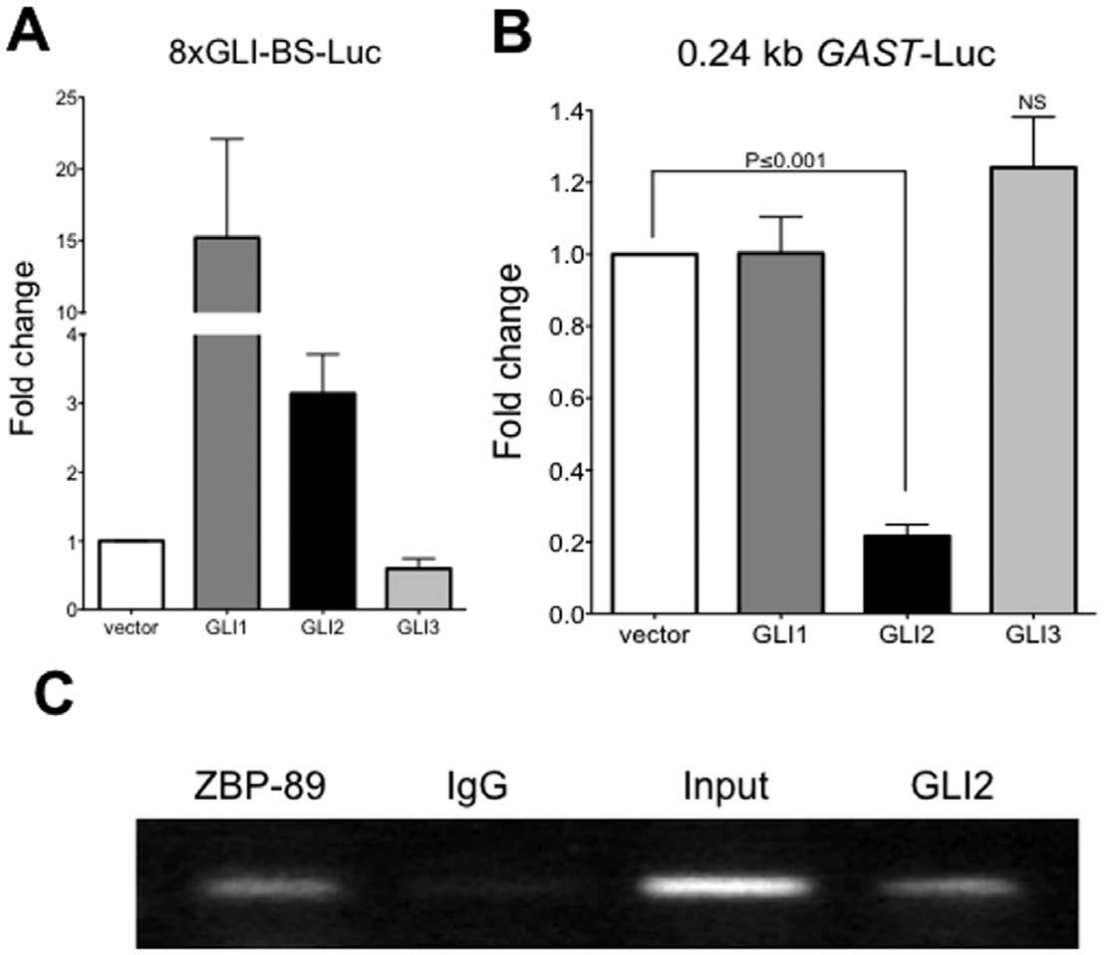
GLI2 repressed *GAST* expression. **A**) AGS cells were co-transfected with GLI1, GLI2 and GLI3 expression vectors and the Shh pathway readout construct (8xGLI-BS-Luc) and tested for promoter activation by luciferase assay. **B**) Luciferase assay of AGS cells co-transfected with the 0.24 kb *GAST*-Luc construct and GLI1, GLI2, and GLI3 expression vectors. **C**) Chromatin immunoprecipitation of AGS cells DNA-protein complexes using anti-ZBP89, rabbit (IgG), and anti-GLI2 antibodies followed by amplification of 216 bp of the proximal gastrin promoter. Data presented as mean±SEM. N = 3 independent experiments with triplicates.

### Quantitative RT-PCR

For mRNA extraction, samples were homogenized in Trizol reagent (Invitrogen, Carlsbad, CA) followed by phenol-chloroform RNA extraction, and purification with the RNeasy Mini Kit (Qiagen, Valencia, CA). First strand cDNA was synthesized using i-script (BioRad, Hercules, CA) according to the manufacturer’s protocol. Triplicates for each sample were amplified by qPCR in a BioRad iCycler using SYBR green. The primer sequences for PCR amplification are shown in Table S1.

### Plasmids, Transfections and Luciferase Assay

All plasmid constructs used in the experiments have been described previously [26], [27], [28], [29], [30]. AGS cells were transiently transfected for 48 h with the indicated plasmids using Lipofectamine 2000 reagent (Invitrogen, Carlsbad, CA), according to the manufacturer’s instructions. Cell lysates were harvested to determine luciferase activity using the Dual-Luciferase Reporter Assay kit (Promega, Madison, WI) and PerkinElmer (Waltham, MA) Wallac Victor3 luminometer. Luciferase activity was normalized to total protein content, which was determined by Bradford colorimetric assay (BCA) Protein Assay Kit (Thermo Scientific, Waltham, MA). The data were expressed as the mean ±SEM for three independent experiments performed in triplicate.

### Chromatin-Immunoprecipitation (ChIP) Assay

The ChIP kit from Millipore (Millipore, Temecula, CA) was according to the manufacturer’s instructions. Briefly, after crosslinking with formaldehyde, AGS cells were collected, lysed then sonicated to shear DNA to an average fragment size of 200–1000 bp. For the “input,” 1% of the lysate was removed for PCR analysis and the remainder was used for immunoprecipitation overnight at 4°C with either rabbit IgG (Santa Cruz Biotechnology, Santa Cruz, CA), rabbit anti-ZBP-89 [31] or anti-GLI2 antibodies (Abcam, Cambridge, MA). The crosslinking was reversed and DNA was recovered using QIAquick PCR Purification Kit (Qiagen, Valencia, CA). The following primers were used to amplify 216 bp of the proximal gastrin promoter (from 5′−218 and 3′−2 bp upstream of the transcriptional start site): forward 5′-GCTCCAGCCCCTCACCATGAAG-3′; reverse 5′-TTGATGCTCCAGGCCTGCCTTA-3′.

### Western Blot

Cells were treated with IL-1β after serum starvation. Proteins were lysed using RIPA buffer (Sigma-Aldrich), quantified using the BCA Protein Assay Kit (Thermo Scientific) and then resolved in SDS-PAGE gels. After transfer to PVDF membranes, the membrane was blotted with primary antibodies overnight for anti-GLI2 (Abcam) and for 1 h with anti-GAPDH (Santa Cruz Biotechnology). Peroxidase-labeled seconday antibodies (Santa Cruz Biotechnology) were incubated with the membranes for an additional hour then bands were visualized using the Supersignal West Pico chemiluminescent substrate (Thermo Scientific).

### Statistics

Gene expression was normalized to HPRT, and changes were calculated using the 2-ΔΔC(T) method [32]. Data are presented as the mean ± S.E.M., and were analyzed by one-way ANOVA, Mann-Whitney or Student’s-*t* tests using Prism software (GraphPad Software, Inc., La Jolla, CA, USA). P values ≤0.05 were considered significant.

**Figure 5:**
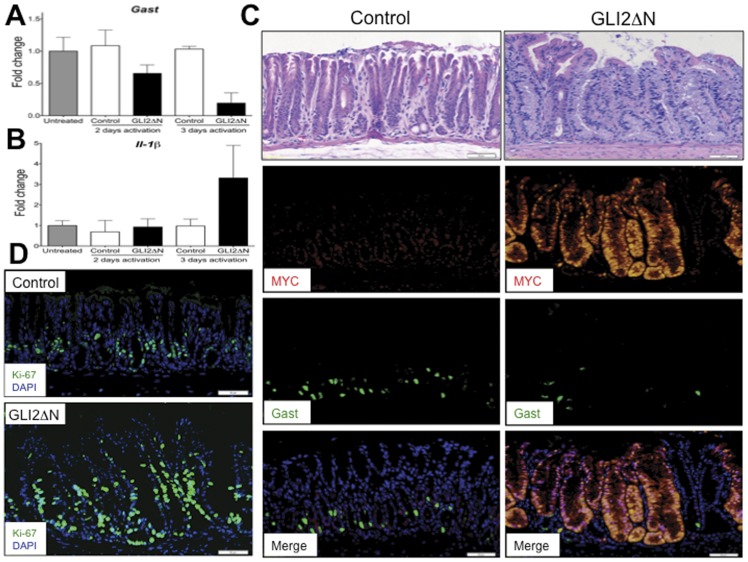
Epithelial activation of Gli2 induced Il-1β and reduced *Gast* expression and G-cell number. **A**) *Gast* gene expression is reduced after Gli2 activation in *Shh-Cre;R26-LSL-rtTA;tetO-GLI2*ΔN (GLI2ΔN) mice after 3 days of treatment with doxycycline. **B**) *Il-1β* was induced in the antra of GLI2ΔN mice antra. **C**) Representative images of hematoxylin-eosin staining (top panels), MYC staining to detect epitope-tagged GLI2?N (red), Gast staining (green) and merged images (lower panel) of the antrum of control and GLI2ΔN mice after 3 days of doxycycline. **D**) Representative images of proliferation marker Ki-67 staining in control and GLI2ΔN mice after 3 days of doxycycline. Data presented as mean±SEM. N = 2 mice per group per time. Bars are 100 µm in panel **C)** and 50 µm in panel **D)**.

## Results

To better define the early mechanisms involved in gastric antral transformation, we studied the antra of 9–13 month-old *Gast^−/−^* mice that showed hyperplastic epithelium but not frank tumors (Fig. 1A–F). The contribution of Hh signaling during development of antral hyperplasia was assessed using three LacZ reporter mice bred onto the *Gast^−/−^* mouse genetic background. *Shh^LacZ^* reporter mice confirmed the restricted epithelial expression of *Shh* to corpus and antral glands. Collectively, we observed a reduction in antral *Shh* expression (P = 0.03) in the *Gast^−/−^* mice compared to their WT littermates (Fig. 1A, B and G). Analysis of reporter mice for *Gli1* expression (*Gli1^LacZ^*) showed *Gli1* expression restricted to stromal cells in both the normal (*Gli1^LacZ^*;*Gast^+/+^*) and hyperplastic antra (*Gli1^LacZ^*;*Gast^−/−^*). Moreover, *Gli1* expression tended to decrease in the antral hyperplastic regions (P = 0.14), suggesting a reduction of Hh signaling in the *Gast^−/−^* antra (Fig. 1C, D and H). The *Gli2^LacZ^*;*Gast^−/−^* mice showed both mesenchymal and epithelial *Gli2* expression, in contrast to exclusively mesenchymal expression in WT mouse antra (Fig. 1E and F). The expression of *Gli2* in the *Gast^−/−^* stomach trended higher, although the expression levels did not achieve statistical significance (P = 0.06) (Fig. 1I). We observed nuclear (yellow arrows, Fig. 1F insert) and perinuclear (black arrows, Fig. 1F insert) β-gal staining in the epithelial cells exhibiting the highest Gli2-LacZ expression along with cytoplasmic accumulation. These results suggested that the increased *Gli2* expression in the antral epithelium of the *Gast^−/−^* mouse was not the result of elevated Shh ligand expression and Hh canonical signaling.

The adjacent corpi of the *Gast^−/−^* mice showed no hyperplastic or other significant histological changes (Fig. 2). However, *Shh^LacZ^* expression in the corpi of *Gast^−/−^* mice was lower than that of the *Gast^+/+^* mice (Fig. 2A and B), accounting for the significant reduction in *Shh* mRNA expression (Fig. 1G) and consistent with the profound hypochlorhydria as previously reported [6]. Expression in the *Gli1^LacZ^* (Fig. 2C and D) and *Gli2^LacZ^* mice (Fig. 2E and F) trended slightly lower in the *Gast^−/−^* corpi (Fig. 2D and F) compared to *Gast^+/+^* (Fig. 2C and E) mice. In contrast to expression in the antrum (Fig. 1F), we did not observe changes in the *Gli2^LacZ^ Gast^−/−^* mouse corpi (Fig. 2F), where *Gli2^LacZ^* expression was restricted to the mesenchyme, suggesting differential regulation of *Gli2* gene expression in the corpus compared to the hyperplastic antrum.

Since inflammatory cytokines, i.e. Il-1β [6], Il-6 [16] and Il-11 [21] have been associated with development of gastric tumors, we analyzed the hyperplastic antra of *Gast^−/−^* mice for the proinflammatory cytokines. *Il-1β*, *Il-6*, *Il-11* and *Infγ* mRNA expression tended to increase in the *Gast^−/−^* antra, achieving statistical significance for *Il-1β* (P = 0.006) and *Il-11* (P = 0.04) (Fig. 3A). To determine if the observed increase in antral *Gli2* expression in the *Gast^−/−^* epithelium could be due to inflammation, the AGS human gastric cell line was treated with IL-1β. IL-1β induced a significant increase in *GLI2* (P = 0.02) (Fig. 3B), while *GLI1* mRNA expression decreased (P = 0.01) (Fig. 3C) further supporting the concept that *GLI2* expression in gastric epithelial cells can be modulated in a Hh-independent manner. Treatment with IL-1β also induced GLI2 expression in the gastric cell line NCI-N87 (Fig. S1), which exhibits characteristics of epithelial cells in the deep antral glands [33]. These results demonstrated that *GLI2* gene expression can be induced in gastric cells by proinflammatory cytokines.

It has been reported that gastrin promotes the development of gastric cancer [34], [35]. Specifically, Datta *et al*. reported that *GAST* mRNA expression can be repressed by IL-1β via Smad7 or NFκB activation [36], [37]. Therefore we tested whether IL-1β suppresses *GAST* gene expression. Treating AGS cells with IL-1β, which express but do not secrete gastrin [38], confirmed that IL-1β does indeed suppress *GAST* mRNA expression (P = 0.001) (Fig. 3D). In the *Gast^−/−^* hyperplastic antrum, the expanded epithelial expression of *Gli2* occurred in the lower portion of the antral gland below the proliferative area, where gastrin-expressing cells are normally located. Since we showed that IL-1β stimulates *GLI2* gene expression but reduces *GAST* expression, we tested the possibility that GLI2 might mediate IL-1β repression of *GAST*.

We co-transfected the expression vectors for GLI1, GLI2 and GLI3 with the 0.24 kb gastrin luciferase reporter (*GAST*-Luc) or the control GLI-responsive reporter plasmid 8xGli-BS-Luc (Fig. 4A) into AGS cells. Indeed, we observed a 50 percent decrease (P = 0.001) in *GAST* promoter activity when transfected with GLI2, but not GLI1 or GLI3, suggesting a GLI2-specific transcriptional regulatory effect on the *GAST* promoter (Fig. 4B). To determine if GLI2 directly binds the *GAST* promoter, we performed chromatin immunoprecipitation (ChIP) for GLI2 in AGS cells and found that GLI2 binds the proximal human gastrin promoter (Fig. 4C). In particular, GLI2 bound to the *GAST* promoter to a similar extent as ZBP-89 (Fig. 4C), another zinc finger protein that we have previously identified as a transcriptional repressor of the *GAST* promoter through a GC-rich element in the proximal promoter [31].

Next we considered the possibility that Gli2 might repress *Gast* gene expression *in vivo*, and thus induce a phenotype similar to that observed in the *Gast^−/−^* mice. To test this hypothesis, we examined the level of *Gast* gene expression in the *Shh-Cre;R26-LSL-rtTA;tetO-GLI2ΔN* (GLI2ΔN) mice, which conditionally express constitutively-active MYC-tagged GLI2 (*GLI2ΔN*) in the epithelium in the presence of doxycycline. Indeed, *Gast* gene expression (P = 0.056) (Fig. 5A) and the number of gastrin-expressing cells (Fig. 5C, green) decreased in the induced GLI2ΔN mice after only 3 days of doxycycline treatment, while *Il-1β* gene expression tended to increase (P = 0.38) (Fig. 5B). We also observed increased proliferation (Fig. 5D) and distorted gland morphology over the same time period (Fig. 5C top panel). However, changes in *Il-6* or *Il-11* mRNA expression (data not shown) or a significant inflammatory infiltrate was not observed (Fig. 5C, top panel). Therefore, we concluded that epithelial GLI2 activation and Il-1β can induce loss of *Gast* gene expression while increasing proliferation, leading to dysplastic changes in the gastric antrum.

## Discussion

Hh signaling is important for maintenance of the gastric mucosa [10], [11]. However it remains unclear whether deregulation of the Hh signal leads to preneoplastic changes and eventually gastric cancer. Therefore, the goal of our study was to determine if Hh signaling contributed to early preneoplastic changes in the antrum where the etiology of gastric cancers has not been well-established. Normal *Shh* expression is highest in the corpus and decreases in the antrum [9]. In addition, expression during Helicobacter infection also reduces ligand expression especially in the corpus [6]. It is important to note that we did not observe histological changes in the corpus when Hh signaling was examined on a *Gast^−/−^* background in which the stomach was hypochlorhydric [6], [39]. Consistent with this finding there was a decrease in *Gli1* expression, in the absence of an obvious inflammatory infiltrate. However in contrast to the corpus, *Gli2* reporter expression on the *Gast^−/−^* genetic background was increased in the epithelial cells of the deep antral glands where hyperplastic changes were also observed. Due to the dissociation between *Shh* and *Gli1* compared to *Gli2* expression, we concluded that the epithelial expression of *Gli2* was likely Shh-independent.

The epithelial-specific expression of constitutively activated GLI2 (GLI2ΔN) *in vivo* proved to be sufficient to induce the loss of *Gast* gene expression and to induce *Il-1β* expression and antral hyperplasia. Our *in vitro* data demonstrated that IL-1β induces *GLI2* expression in epithelial gastric cells, while *in vivo* GLI2ΔN activation resulted in a significant induction of *Il-1β* expression, similar to what has been reported in the skin of mice expressing Gli2 [40]. However, whether there is reciprocity between *Gli2* and *Il-1β* expression requires further investigation.

Antral tumorigenesis in the *Gast^−/−^* mice has been associated with bacterial overgrowth [19] and inflammation [20], [21]. Our previous report on antral tumors in the *Gast^−/−^* mice showed that increased expression of *Il-1β*, of the Tgfβ -family member activin A (AcA) and follistatin (Fst), the bmp/activin antagonist, preceded transformation in the *Gast^−/−^* mice antrum [18], suggesting that there are multiple signal transduction pathways that contribute to the development of gastric cancer. There are different reports supporting the possible interactions. First, IL-1β is known to induce the expression of AcA in different cell types [41], [42]. Second, there is evidence of a strong link between TGFβ signaling and GLI2, such that TGFβ-activation of the Smad signaling cascade inducing *GLI2* expression [43], [44]. This induction has been shown to be important for cancer development in organs other than the stomach [45], [46], [47]. Furthermore, Gli2 has been shown to induce the expression of *Fst* [48], which probably serves as a negative feedback in response to the increase in activins or BMPs.

In the present study, we focused on the early changes observed in hyperplastic antra of *Gast^−/−^* mice between 9 and 13 months of age that had no evidence of histological changes in the corpus. The difficulty of analyzing early lesions is the limited ability to achieve statistical differences for some markers that have been shown to be important in gastric cancer development. This is especially important for the observed increase in *Il-6* expression that did not reach statistical significance in our study, but has previously been reported to be important for tumor formation in the stomach [21]. Interestingly, we consistently observed significant increases in *Il-11* mRNA, suggesting that this cytokine is of importance in the development of antral tumors, and might participate in the regulation of parietal cell function and acid secretion in the corpus [49].

Our novel finding of Gli2 being a regulator of *Gast* expression potentially complements the previous work by Datta *et al*. showing that both TGFβ and IL-1β negatively regulate *GAST* expression [36]. However, our previous report suggests that the factors involved in antral changes are the Tgfβ-related molecules AcA and its inhibitor Fst [18]. Although the mechanisms triggering AcA expression and initiating *Gli2* and *Il-1β* induction remain to be defined, bacterial overgrowth in the hypochlorhydric *Gast^−/−^* stomach is a potential culprit. When Jones *et al*. challenged mice with LPS, they observed a rapid increase in circulating AcA mediated by TLR4 activation, that was soon followed by increased levels of circulating Fst, Il-6 and Il-1β [50]. It is thus conceivable that gastric bacteria stimulate AcA and Il-1β expression, which in turn induces Gli2 in gastric epithelial cells, leading to Fst expression as a negative feedback and a reduction of *Gast* expression, further altering gastric homeostasis.

Overall, our study provides evidence of inflammation-driven Hh-independent induction of Gli2 in the gastric epithelium and indicates that Gli2 is a direct negative regulator of *Gast*. As a result, inflammatory mediators, such as Il-1β, Il-11 and AcA, along with epithelial Gli2, appear to be important epithelial drivers of the histologic changes during antral transformation.

## Supporting Information

Figure S1 IL-1β induces GLI2 expression in NCI-N87 cells. The gastric cell line NCI-N87 was treated with different doses of IL-1β for 24 hr. Protein was resolved by SDS-PAGE, transferred to PVDF membrante and then blotted for GLI2 and GAPDH as the loading control.
(TIF)

Table S1 Primer sequences.
(DOCX)
