# Supplementary figures and images for: Inflammation and Gli2 Suppress Gastrin Gene Expression in a Murine Model of Antral Hyperplasia
Source: PLoS One. 2012 Oct 24;7(10):e48039. doi: 10.1371/journal.pone.0048039 (PMC3480483; doi:10.1371/journal.pone.0048039)

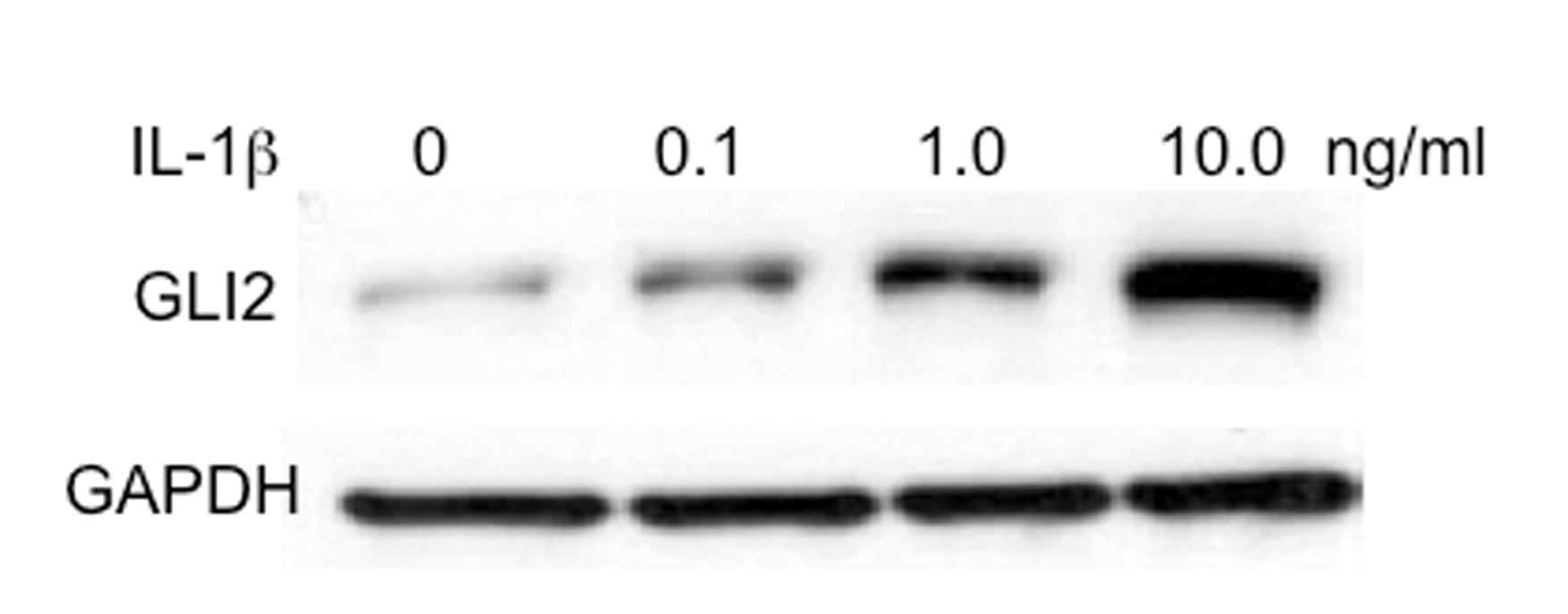

Supplement: Figure S1 — IL-1β induces GLI2 expression in NCI-N87 cells. The gastric cell line NCI-N87 was treated with different doses of IL-1β for 24 hr. Protein was resolved by SDS-PAGE, transferred to PVDF membrante and then blotted for GLI2 and GAPDH as the loading control. (TIF) [file pone.0048039.s001.tif]
