# Supplementary material for: Inflammation and Gli2 Suppress Gastrin Gene Expression in a Murine Model of Antral Hyperplasia
Source: PLoS One. 2012 Oct 24;7(10):e48039. doi: 10.1371/journal.pone.0048039 (PMC3480483; doi:10.1371/journal.pone.0048039)
Supplement: Table S1 — Primer sequences. (DOCX) [file pone.0048039.s002.docx]

**Table S1**. Primer sequences.

| Gene | Forward Primer | Reverse Primer |
| --- | --- | --- |
| mGastrin | ACACAACAGCCAACTATTC | CAAAGTCCATCCATCCGTAG |
| mShh | ATGTTTTCTGGTGATCCTTGCT | ATCGTTCGGAGTTTCTTGTGAT |
| mGli1 | TTG GGA TGA AGA AGC AGT TG | GGA GAC AGC ATG GCT CAC TA |
| mGli2 | ACCCAACACTCAGCAGCAGTAGC | GCTCCGCTTATGAATGGTGATGG |
| mHPRT | AGG ACC TCT CGA AGT GTT GGA TAC | AAC TTG CGC TCA TCT TAG GCT TTG |
| mIL-1β | CAACCAACAAGTGATATTCTCCATG | GATCCACACTCTTCCAGCTGCA |
| mIL-6 | GAGGATACCACTCCCAACAGACC | AAGTGCATCATCGTTGTTCATACA |
| mIL-11 | CTG CAAGCCCGACTGGA | AGGCCAGGCGAGACATCA |
| mIFNγ | TCAAGTGGCATAGATGTGGAAGAA | TGGCTCTGCAGGATTTTCATG |
| hGli2 | TGGCCGCTTCAGATGACAGATGTTG | CGTTAGCCGAATGTCAGCCGTGAAG |
| hGastrin | GCCCAGCCTCTCATCATC | GCCGAAGTCCATCCATCC |
| hHPRT | AGCAAGACGTTCAGTCCTGTC | CAGCCCTGCCGTCGTGATTA |
| hGli1 | CCCAGCGCCCAGACAGAG | GACAGTCAGGGCTCGCATAG |
| hInbA (AcA) | GGAGGGCAGAAATGAATGAA | ATCTCGAAGTGCAGCGTCTT |

m: mouse

h: human
